# Taxonomic revision of imitating carpenter ants, Camponotus subgenus Myrmopytia (Hymenoptera, Formicidae) of Madagascar, using morphometry and qualitative traits

**DOI:** 10.3897/zookeys.681.13187

**Published:** 2017-06-21

**Authors:** Nicole Rasoamanana, Sándor Csősz, Brian L. Fisher

**Affiliations:** 1 Madagascar Biodiversity Center, BP 6257, Parc Botanique et Zoologique de Tsimbazaza, Antananarivo, Madagascar; 2 Entomology, California Academy of Sciences, 55 Music Concourse Drive, San Francisco, CA 94118, U.S.A.

**Keywords:** Malagasy region, taxonomic revision, *Camponotus*, subgenus *Myrmopytia*, NUMOBAT, species delimitation, exploratory analyses, biogeography

## Abstract

The ant genus *Camponotus* (Mayr, 1861) is one of the most abundant and species rich ant genera in the Malagasy zoogeographical region. Although this group is commonly encountered, its taxonomy is far from complete. Here, we clarify the taxonomy of the Malagasy-endemic Camponotus subgenus Myrmopytia (Emery, 1920). Species delimitation was based on traditional morphological characters and multivariate morphometric analyses, including exploratory Nest Centroid clustering and confirmatory cross-validated Linear Discriminant Analysis. Four species are recognized: *Camponotus imitator* (Forel, 1891), *Camponotus jodina* **sp. n.**, *Camponotus karaha* **sp. n.**, and *Camponotus longicollis* **sp. n.** All four species appear to mimic co-occurring Aphaenogaster species. A diagnosis of the subgenus Myrmopytia, species descriptions, an identification key based on minor and major subcastes of workers, and the known geographical distribution of each species are provided.

## Introduction

**Figure 1.**
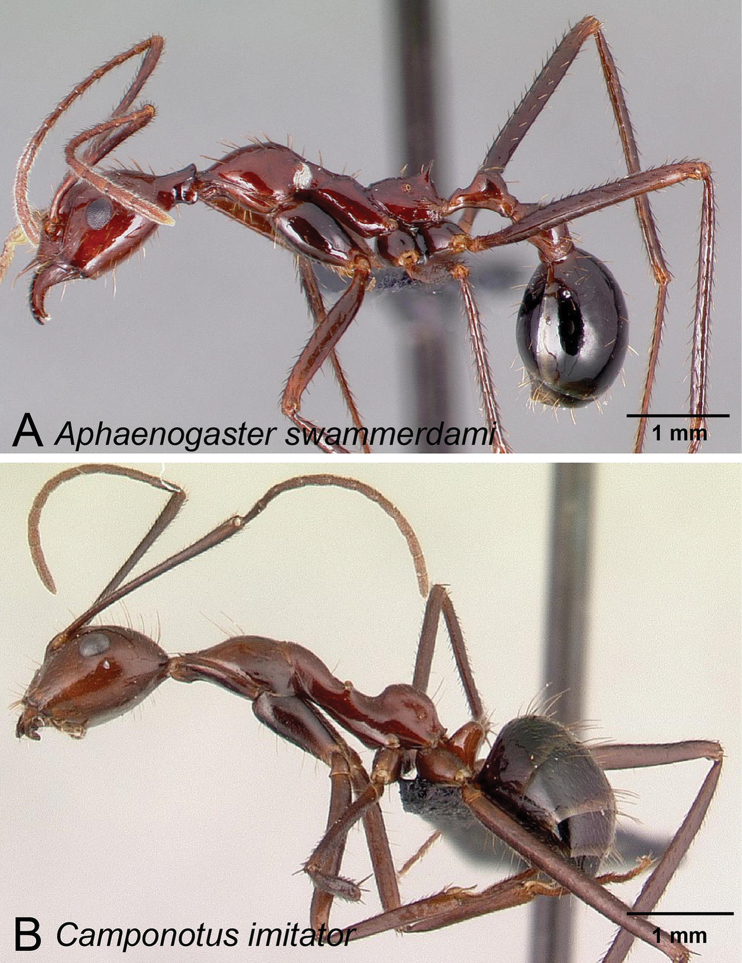
Camponotus species in thesubgenus Myrmopytia are thought to mimic co-occurring Aphaenogaster species **A** *Aphaenogaster swammerdami* (CASENT0017663) **B** *Camponotus imitator* (CASENT0452849).

Malagasy *Camponotus* species are known to mimic other ant species or genera, such as *Tetraponera*, *Catalaucus* and *Aphaenogaster*, highlighting the incredible potential for morphological adaptation in *Camponotus* species (Forel 1886, Ward 2009, BLF, pers. obs.). In this study, we assess the diversity of the Malagasy subgenus Myrmopytia. Minor workers of this subgenus all appear to mimic *Aphaenogaster*. In 1891, when describing *Camponotus imitator*, Forel noted that the minor workers exhibited strikingly similar morphology to the myrmicine ant *Aphaenogaster swammerdami* Forel, 1886 (Formicidae: Myrmicinae) (see Figure 1). Little is known about the evolutionary advantage of this phenomenon, although where *A. swammerdami* occurs, it is a dominant species and there might be some level of protection in imitating a dominant ant. The genus *Camponotus* (Mayr, 1861) is among the most diverse and abundant ant lineages in Madagascar. Of the more than 1500 valid species and subspecies worldwide, 78 *Camponotus* species are known from the Malagasy region (Antcat.org) with an additional estimated 100 undescribed taxa (AntWeb.org). Several complications have hindered progress in the refinement of *Camponotus* taxonomy. The first is morphological variation, especially the high level of intraspecific and intranidal polymorphism; the presence at times of three morphological castes (minor, media and major); and the poorly tested diagnostic value of morphological traits that make species delimitation using conventional approaches extremely difficult. The second difficulty is linking past works to current taxon concepts, as these same morphological challenges complicate the association of previously named type specimens to current species hypotheses. The third complication is that the existing subgenus classification of *Camponotus* is an amalgamation of past taxonomic works that have tried to “fix” the problem of taxa delimitation by adding new names rather than by completely revising the global classification (Bolton 1995, Clouse et al. 2015, Ward et al. 2016).

We have attempted to overcome the problem of morphological variation in the Malagasy-endemic Camponotus subgenus Myrmopytia (Emery, 1920) by combining traditional morphology (i.e. evaluation of qualitative traits) with a numeric morphology-based approach. For quantitative analyses of morphometric data we follow the protocol introduced by Csősz and Fisher (2016) using a combination of NC-clustering (Seifert et al. 2014) and PART (Nilsen and Lingjaerde 2013). In this protocol, boundaries of operational taxonomic units (OTUs) are tested via cross-validated Linear Discriminant Analysis (LOOCV-LDA). In order to obtain high quality resolution of morphometric analyses we extended the existing set of morphometric characters by including many new traits. Our extended character set includes 19 traits of which four are defined for the first time.

In terms of overcoming the challenges of historical names, our work builds on 15 years of tracking down type material of all Malagasy taxa from across European collections to assess the identity of previous descriptions in relation to more recently collected material. Although we are still missing a small percentage of *Camponotus* type specimens, the identities of most names have been clarified.

Lastly, this work does not address the issue of the unnatural subgeneric classification for the genus which is better addressed with molecular phylogenetic methods and global sampling. Instead, for this revision, we retain the historic subgenus Myrmopytia, for *Camponotus imitator* (Forel, 1891), and include for the convenience of this revision, species with morphologically similar workers.

All species are described here based on the worker caste, and the key provided combines qualitative characters and morphometric ratios that will help resolve the most problematic cases. Distribution maps are also provided.

## Materials and methods

### Abbreviations of depositories

**CASC** California Academy of Sciences, San Francisco, CA, USA.

**MHNG** Muséum d’Histoire Naturelle, Geneva, Switzerland.

**NHMB** Naturhistorisches Museum, Basel, Switzerland.

**PBZT** Parc Botanique et Zoologique de Tsimbazaza, Antananarivo, Madagascar.

**PSWC** P. S. Ward Collection, University of California, Davis, CA, USA.

**ZMHB** Museum für Naturkunde, Humboldt-Universität, Berlin, Germany.

### Materials

The morphometric analysis was based on 130 workers principally collected by B.L. Fisher and the Madagascar Biodiversity Center team in 74 collecting events across Madagascar. Specimen data and images for material examined in this study is available on AntWeb (http://www.antweb.org) and can be accessed using the unique specimen identifiers (e.g. CASENT0101365).

### Methods

**Imaging**

Digital color montage images were created using a JVC KY-F75 digital camera and Syncroscopy Auto-Montage software (version 5.0), or a Leica DFC 425 camera in combination with the Leica Application Suite software (version 3.8).

**Mapping**

Distribution maps for all species were generated by importing specimen distribution records into the Diva-GIS program (Hijmans et al. 2011). For material lacking locality data such as older type material, georeferenced coordinates are placed in brackets. Georeferencing was completed with the aid of online maps and the Missouri Botanical Garden’s Gazetteer to Malagasy Botanical Collecting Localities (2015).

### Morphometric character recording and terminology

Measurements were taken with a Leica MZ 9.5 stereomicroscope equipped with a cross-scaled ocular micrometer. Each worker was evaluated using 19 continuous morphometric traits, measured as in Rakotonirina et al. (2016) for the *Camponotus edmondi* group. The morphometric data are expressed in µm. All measurements were made by NR allowing to achieve the highest consistency in character recording throughout the entire data collection procedure.

**Figure 2.**
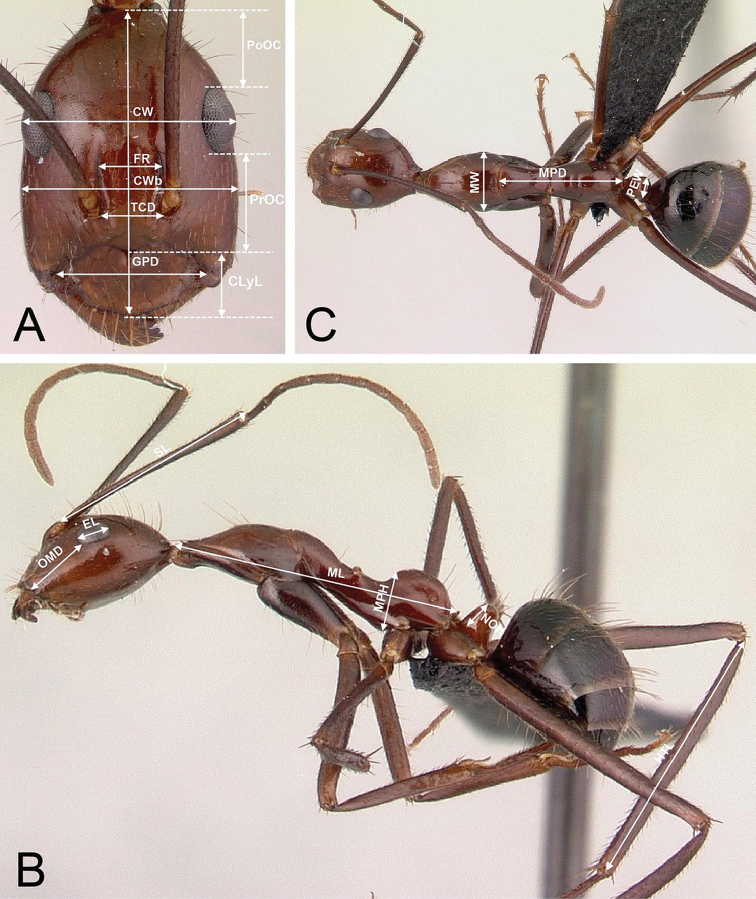
Illustrations of measurements used to distinguish members of *Myrmopytia*. **A** Full-face view of the head illustrating measurement lines for maximum cephalic length (CL), clypeal length (ClyL), maximum cephalic width (CW), maximum head capsule width (CWb), eye length (EL), frontal carina distance (FR), maximum tentorial pit distance (GPD), postocular distance (PoOC), preocular distance (PrOC) and torular carina distance (TCD) **B** Lateral view of the body illustrating measurement lines for oculo-mandibular distance (OMD), scape length (SL), mesosoma length (ML), mesothoracico-propodeal height (MPH), petiolar node height (NOH) and maximum hind tibia length (HTL) **C** Dorsal view of the body illustrating measurement lines for mesosoma width (MW), mesothoracico-propodeal distance (MPD) and petiolar width (PEW).

Definitions and abbreviations for the measured characters are as follows (see Figure 2):

**CL** *Maximum cephalic length*. The maximum median length of the head in full-face view, measured from the midpoint of the posterior margin of head to the midpoint of the anterior margin of the clypeus (Fig. 2A).

**ClyL** *Clypeal length*. The maximum median length of the clypeus measured from the posterior margin to the anterior margin in frontal view, in which the anterior and posterior clypeal margins are aligned to the same focus. Median concavity on either or both margins reduces the length of the clypeus (Fig. 2A).

**CS** *Cephalic size*. This derived character is used as body size indicator and is calculated from the arithmetic mean of head length (CL) and maximum head capsule width (CWb).

**CW** *Maximum cephalic width*. The longest distance between the lateral margins of the compound eyes in full-face view (Fig. 2A).

**CWb** *Maximum head capsule width*. The maximum width of the head capsule excluding the compound eyes (Fig. 2A).

**EL** *Eye length*. Maximum diameter of the compound eye (Fig. 2A).

**FR** *Frontal carina distance*. The longest distance between the frontal carinae (Fig. 2A).

**GPD** *Maximum tentorial pit distance*. The longest distance between the centers of the fossae located at or very close to the posterolateral margin of the clypeus (Fig. 2A).

**HTL** *Maximum hind tibia length*. Straight line length of the hind tibia measured from the constriction immediately before its proximal insertion to its distalmost point, excluding the bristles or spines (Fig. 2B).

**ML** *Mesosoma length*: The longest anatomical line that connects the posteriormost point of the propodeal lobe with the anteriormost point of the pronotal collar; preferentially measured in lateral view, but if one of the reference points is not visible, dorsal view may help (Fig. 2B).

**MPD** *Mesothoracico-propodeal distance*. With the promesonotal suture and the anterior petiolar foramen margin in the same plane of focus in dorsal view, the maximum length between the promesonotal suture and the posteriormost point of the propodeal process dorsal to the petiolar insertion (Fig. 2C).

**MPH** *Mesothoracico-propodeal height*. Measured in lateral view, using as a diagonal reference line that connects the anteriormost point of the pronotal shield and the posteriormost point of the propodeal process dorsal to the petiolar insertion, MPH is the perpendicular distance between two lines parallel to this line, one of which just touches the anteroventral corner of the mesopleuron, dorsal to the insertion of the mesocoxa, and other which the dorsalmost point of the propodeum (Fig. 2B).

**MW** *Mesosoma width*. Maximum width of the pronotum in dorsal view, which in the *Myrmopytia* is also the maximum mesosomal width (hence “mesosoma width”) (Fig. 2C).

**NOH** *Petiolar node height*. The maximum distance between the petiolar spiracle and the dorsalmost point of the petiolar node (Fig. 2B).

**OMD** *Oculo-mandibular distance*. The minimum distance between the anterior margin of the compound eye and the mandibular insertion to the head (Fig. 2B).

**PEW** *Petiolar width*. The maximum width of the petiole in dorsal view (Fig. 2C).

**PoOC** *Postocular distance*. The longest distance between the posteromedian margin of the head and the reference line set on the posterior margins of the two compound eyes. Measured at a right angle to the reference line in full-face view (Fig. 2A).

**PrOc** *Preocular distance*. The longest distance between the anteromedian margin of the clypeus and the level of the anterior margin of the compound eyes as reference line. Measured at a right angle to the reference line in full-face view (Fig. 2A).

**SL** *Scape length*. Straight line length of the first antennal segment excluding the basal condyle (Fig. 2B).

**TCD** *Torular carina distance*. The minimum distance between the torular arches that surround the antennal insertion (Fig. 2A).

### Morphometric data analysis

Altogether 130 worker individuals (96 minor and 34 major workers) were measured and analyzed using multivariate statistics based on the protocol described by Seifert et al. (2014) and extended by Csősz and Fisher (2016). *Camponotus* species may produce two to three subcastes of workers exhibiting quite different allometric properties. The Linear Discriminant Analysis (LDA) cannot properly analyze non-linearly scaled data. Therefore, all specimens were sorted into two subcastes (minor and major were found in the present subgenus) via visual inspection of scaling properties using pair-wise matrix scatterplots (see also Rakotonirina et al. 2016). With this procedure, linear within-class allometries were achieved for each trait.

Thanks to the higher number of minor workers (96 were minors out of 130 total) this subcaste was selected for the purpose of morphometric data analyses. Morphometric data and descriptions for major workers are also provided, but their morphometric data were not included in statistical processing.

Generation of species hypotheses by combined application of NC-PART clustering and confirmatory Linear Discriminant Analysis (LDA)

We evaluated the morphometric data recorded from 96 minor workers belonging to 66 samples. All samples represent nest series or individuals from one collecting event. We used a combined application of NC-clustering (Seifert et al. 2014) and PART algorithm implemented in ‘clustergenomics’ package (Nilsen and Lingjaerde 2013) under R environment (R Core Team 2014).

The optimal number of clusters and the partitioning of samples done by Partitioning Based on Recursive Thresholding (PART) were accepted as improved species hypothesis worth testing if the two clustering methods, ‘hclust’ and ‘kmeans’ via PART arrived at the same conclusion (see Seifert et al. 2014, Rakotonirina et al. 2017).

Confirmatory Linear Discriminant Analysis with leave-one-out cross-validation (LOOCV-LDA) was run to confirm species boundaries. The classification returned by the quantitative analyses were further verified by searching for qualitative differences of morphological traits between clusters.

## Results and discussion

**Figure 3.**
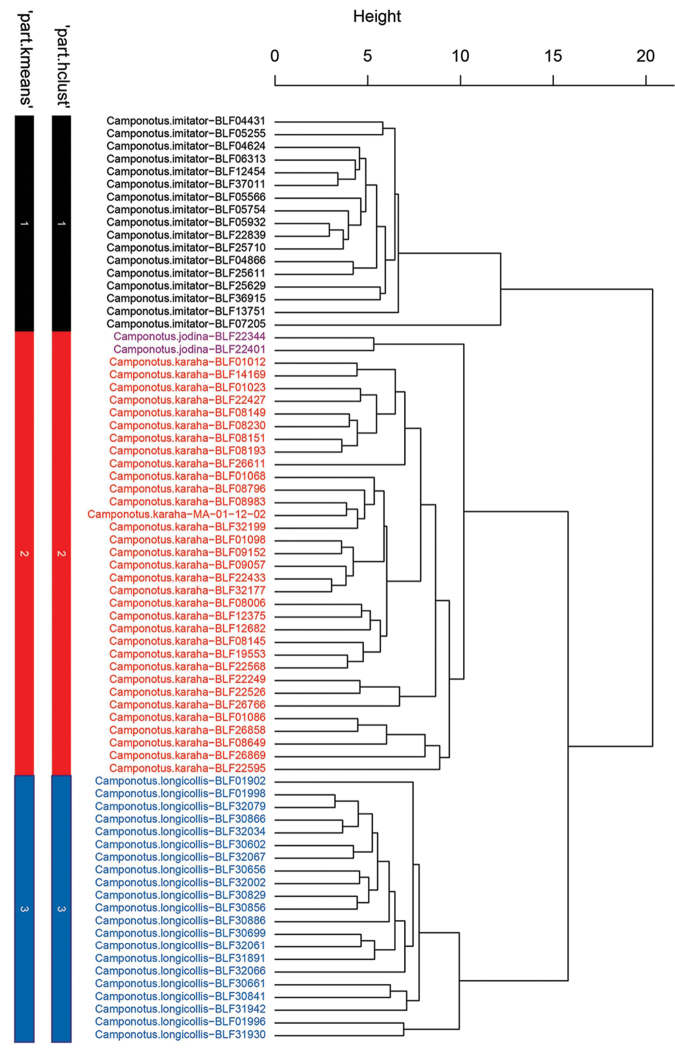
Dendrogram solution for the Malagasy Myrmopytia based on morphometric data. Labels include final species hypothesis followed by collection code separated by a hyphen. The color bars show partitioning yielded by two cluster methods ‘hclust’ and ‘kmeans’ via method PART. Color code: *Camponotus imitator*: black (cluster 1), *C. jodina*: purple (cluster 2), *C. karaha*: red (cluster 2), *C. longicollis*: blue (cluster 3)

The NC-PART clustering found three clusters (see numbered bars 1 (black), 2 (red) and 3 (blue) in Figure 3). Qualitative investigation of morphological traits found overall support for these three clusters. However, in cluster 2 (red bar in Figure 3) two samples remarkably differ in morphology from the other specimens in the cluster. Based on the distinct phenotype of the two individuals of the satellite cluster (purple specimens in Figure 3), we propose that these two individuals represent an additional species. These individuals also appear slightly separated in the NC-clustering dendrogram. A larger sample size, however, would be required to statistically evaluate the independence of the cluster using ‘kmeans’ or ‘hclust’. Based on the morphometric and qualitative analysis, four species are hereby named as *C. imitator* Forel, 1891, *C. longicollis* sp. n., *C. karaha* sp. n. and *C. jodina* sp. n. (see Figure 3).

The confirmatory Linear Discriminant Analysis with leave-one-out cross-validation (LOOCV-LDA) yielded complete classification success and every specimen was correctly classified to one of the four species. It is important to note that even though the two specimens of *C. jodina* were correctly classified, the small sample size (2 specimens) is below the sample size criterion for LDA. As supporting evidence for *C. jodina*, univariate comparison of body ratios, PEW/CS and MPD/CS of samples of *C. jodina* and the most similar species, *C. karaha* yield non-overlapping ranges (Table 1).

**Table 1.** Ratios of morphometric data for minors and majors of the four species. The means of the ratios and the standard deviations are written in bold; minimum and maximum values are shown in parentheses. CS (Cephalic size) values are given in millimeters.

Species	Worker castes	CS	CWb/CL	CW/CL	PoOC/CL	PrOC/CL	ClyL/CL	FR/CS	TCD/CS	GPD/CS	SL/CS
*imitator*	Minors (n=25)	**1.68±0.2**	**0.76±0.0**	**0.73±0.0**	**0.25±0.0**	**0.54±0.0**	**0.27±0.0**	**0.25±0.0**	**0.23±0.0**	**0.47±0.0**	**1.52±0.1**
		[1.34, 2.18]	[0.71, 0.85]	[0.68, 0.78]	[0.22, 0.29]	[0.51, 0.57]	[0.23, 0.29]	[0.21, 0.28]	[0.16, 0.25]	[0.42, 0.51]	[1.21, 1.73]
	Majors (n=22)	**3.26±0.5**	**0.95±0.0**	**0.79±0.0**	**0.28±0.0**	**0.53±0.0**	**0.29±0.0**	**0.26±0.0**	**0.23±0.0**	**0.39±0.0**	**0.85±0.1**
		[2.32, 3.94]	[0.84, 1.03]	[0.76, 0.84]	[0.24, 0.32]	[0.42, 0.56]	[0.27, 0.32]	[0.25, 0.28]	[0.21, 0.24]	[0.37, 0.42]	[0.71, 1.16]
*jodina*	Minors (n=2)	**1.84±0.0**	**0.53±0**	**0.56±0**	**0.41±0**	**0.40±0.0**	**0.22±0**	**0.22±0**	**0.18±0**	**0.40±0**	**1.87±0**
		[1.80, 1.88]	[0.52, 0.54]	[0.55, 0.57]	[0.40, 0.42]	[0.40, 0.41]	[0.21, 0.23]	[0.22, 0.23]	[0.17, 0.19]	[0.40, 0.40]	[1.85, 1.88]
	Majors unknown	–	–	–	–	–	–	–	–	–	–
*karaha*	Minors (n=47)	**1.72±0.1**	**0.54±0**	**0.58±0**	**0.43±0**	**0.39±0**	**0.22±0**	**0.23±0**	**0.19±0**	**0.41±0**	**1.83±0**
		[1.35, 1.92]	[0.49, 0.60]	[0.56, 0.61]	[0.40, 0.47]	[0.35, 0.43]	[0.18, 0.24]	[0.21, 0.26]	[0.17, 0.22]	[0.37, 0.46]	[1.65, 2.05]
	Majors (n=9)	**2.10±0.1**	**1.47±0**	**1.47±0**	**0.38±0**	**0.43±0**	**0.26±0**	**0.27±0**	**0.21±0**	**0.41±0**	**1.30±0**
		[1.92, 2.28]	[1.39, 1.55]	[1.42, 1.55]	[0.36, 0.39]	[0.41, 0.45]	[0.24, 0.28]	[0.25, 0.29]	[0.20, 0.22]	[0.38, 0.42]	[1.22, 1.41]
*longicollis*	Minors (n=22)	**2.03±0.1**	**0.55±0**	**0.56±0**	**0.42±0**	**0.40±0**	**0.23±0**	**0.21±0**	**0.18±0**	**0.41±0**	**1.60±0**
		[1.74, 2.26]	[0.52, 0.59]	[0.54, 0.58]	[0.36, 0.47]	[0.38, 0.43]	[0.21, 0.26]	[0.19, 0.22]	[0.16, 0.20]	[0.39, 0.44]	[1.43, 1.68]
	Majors (n=3)	**2.42±0.0**	**1.53±0**	**1.59±0**	**0.39±0**	**0.43±0**	**0.27±0**	**0.24±0**	**0.19±0**	**0.40±0**	**1.31±0**
		[2.41, 2.44]	[1.46, 1.60]	[1.54, 1.63]	[0.39, 0.40]	[0.43, 0.45]	[0.25, 0.27]	[0.24, 0.25]	[0.18, 0.21]	[0.39, 0.41]	[1.26, 1.34]
**Species**	**Worker castes**	**MW/CS**	**PEW/CS**	**ML/CS**	**MPD/CS**	**NOH/CS**	**HTL/CS**	**OMD/CS**	**EL/CS**	**MPH/CS**	**ML/MPH**
*imitator*	Minors (n=25)	**0.59±0.0**	**0.24±0.0**	**1.96±0.1**	**1.18±0.1**	**0.24±0.0**	**2.08±0.2**	**0.47±0.0**	**0.24±0.0**	**0.49±0.0**	**4.04±0.3**
		[0.57, 0.63]	[0.16, 0.26]	[1.67, 2.15]	[0.95, 1.40]	[0.18, 0.27]	[1.71, 2.39]	[0.33, 0.51]	[0.20, 0.27]	[0.46, 0.53]	[3.64, 4.63]
	Majors (n=22)	**0.54±0.0**	**0.22±0.0**	**1.34±0.1**	**0.85±0.1**	**0.19±0.0**	**1.23±0.2**	**0.42±0.0**	**0.18±0.0**	**0.41±0.0**	**3.28±0.3**
		[0.52, 0.56]	[0.20, 0.25]	[1.17, 1.66]	[0.69, 1.10]	[0.11, 0.22]	[1.01, 1.64]	[0.40, 0.45]	[0.16, 0.21]	[0.37, 0.47]	[2.84, 3.88]
*jodina*	Minors (n=2)	**0.52±0**	**0.15±0**	**2.29±0**	**1.42±0**	**0.25±0**	**2.20±0**	**0.43±0**	**0.21±0**	**0.41±0**	**5.56±0**
		[0.50, 0.53]	[0.14, 0.15]	[2.25, 2.33]	[1.42, 1.42]	[0.23, 0.27]	[2.17, 2.24]	[0.42, 0.44]	[0.21, 0.22]	[0.40, 0.42]	[5.30, 5.83]
	Majors unknown	–	–	–	–	–	–	–	–	–	–
*karaha*	Minors (n=47)	**0.51±0**	**0.19±0**	**2.20±0**	**1.40±0**	**0.20±0**	**2.22±0**	**0.40±0**	**0.22±0**	**0.42±0**	**5.26±0**
		[0.46, 0.55]	[0.16, 0.24]	[1.98, 2.40]	[1.19, 1.59]	[0.16, 0.27]	[1.93, 2.55]	[0.36, 0.45]	[0.20, 0.24]	[0.33, 0.50]	[4.50, 6.66]
	Majors (n=9)	**0.53±0**	**0.22±0**	**1.87±0**	**1.24±0**	**0.17±0**	**1.75±0**	**0.42±0**	**0.21±0**	**0.42±0**	**4.48±0.63**
		[0.46, 0.56]	[0.21, 0.25]	[1.80, 2.00]	[1.10, 1.37]	[0.16, 0.19]	[1.64, 1.93]	[0.40, 0.44]	[0.20, 0.23]	[0.32, 0.45]	[4.08, 6.13]
*longicollis*	Minors (n=22)	**0.58±0**	**0.20±0**	**2.20±0**	**1.47±0**	**0.12±0**	**2.02±0**	**0.42±0**	**0.21±0**	**0.45±0**	**4.86±0**
		[0.54, 0.61]	[0.17, 0.23]	[2.10, 2.28]	[1.34, 1.58]	[0.09, 0.18]	[1.82, 2.14]	[0.37, 0.46]	[0.19, 0.23]	[0.41, 0.53]	[4.04, 5.40]
	Majors (n=3)	**0.62±0**	**0.20±0**	**1.92±0**	**1.30±0**	**0.14±0**	**1.59±0**	**0.39±0**	**0.20±0**	**0.44±0**	**4.32±0**
		[0.58, 0.64]	[0.19, 0.21]	[1.85, 1.99]	[1.27, 1.37]	[0.13, 0.15]	[1.54, 1.68]	[0.38, 0.40]	[0.19, 0.20]	[0.42, 0.46]	[4.14, 4.69]

### Diagnosis of the subgenus Myrmopytia in the Malagasy region

*Myrmopytia*, Emery 1920: 243: *Myrmopytia* as subgenus of *Camponotus* (Mayr, 1861).

**Type-species.** *Camponotus imitator* Forel, 1891: 209, by monotypy.

*Camponotus imitator* was placed in the subgenus Myrmosphincta by Forel (1912). Emery, 1920 placed it in a new subgenus, *Myrmopytia*, based on the elongate form of the mesosoma.

Minor workers of this subgenus can be recognized by the combination of the following characters: maxillary palps particularly long with respect to head length, extending past a point on line with posterior margin of eye; mesosoma slender, mesonotum elongated and constricted at midlength, propodeum a protruding rounded hump; mesothoracic spiracles prominent, pointing upward; propodeum protuberant. The major worker can be recognized by the distinct form of the mesosoma. The propodeum is dome-like or subrectangular in lateral view and the metanotum is elongate and impressed, and the upper mesopleuron is laterally pinched (see Figures 10A, 14A, 17A).

### Synoptic species list of the subgenus Myrmopytia

*Camponotus imitator* Forel, 1891

= Camponotus imitator var. resinicola Santschi, 1911, **syn. n.**

*Camponotus jodina* **sp. n.**

*Camponotus karaha* **sp. n.**

*Camponotus longicollis* **sp. n.**

### Key to species of Camponotus subgenus Myrmopytia

**Table:** 

1	**Minor**: in full-face view, the posterior region of the head elongated into a short, broad neck (Fig. 4A); Post ocular distance shorter: PoOC/CL <0.304. **Major**: head wedge-shaped (Fig. 5A); head wider: CWb/CL >0.718. **Minor and major**: clypeus with a broad rectangular projection; erect to suberect brown pilosity present on entire dorsum; head and mesosoma red, gaster black	***imitator***
–	**Minor**: in full-face view, the posterior region of the head elongated into long, narrow neck (Fig. 4B); Post ocular distance longer: PoOC/CL >0.404. **Major**: head more rectangular or tapering to rear (Fig. 5B); head narrower: CWb/CL <0.717. **Minor and major**: entire anterior clypeal margin produced into an obtuse angle; appressed to subdecumbent white hairs present on its entire dorsum; body uniformly dark brown to black	**2**
2	**Minor and major**: in profile, petiole relatively low and nodiform, with the anterodorsal angle higher than the petiolar spiracle, NOH/CS <0.146 (Fig. 6A); anterior tentorial pit slightly impressed so that the posterior portion of clypeus protrudes weakly in lateral view (Fig. 7A). **Major**: head narrowed posteriorly, dorsal outline of mesonotum distinctly constricted longitudinally, propodeum protuberant	***longicollis***
–	**Minor and major**: in profile, petiole conical, with the anterodorsal angle on the same level as or below the petiolar spiracle, NOH/CS >0.160 (Fig. 6B); anterior tentorial pit distinctly impressed so that posterior portion of clypeus protrudes strongly in lateral view (Fig. 7B). **Major**: head of major worker more rectangular, dorsal outline of mesosoma forms a smooth curve separated by weak promesonotal suture and mesopropodeal impression	**3**
3	**Minor worker**: in lateral view, petiolar apex forms an acute to a right angle, dorsal face of petiole flattened with distinct lateral edge, PEW/CS >0.169 (Fig. 8A); propodeum hemispherical; integument of mesonotum and propodeum glabrous to finely sculptured (Fig. 8A); antennal funiculus, basitarsi and tarsi yellowish to light brown	***karaha***
–	**Minor worker**: in lateral view, petiolar apex forms a blunt spine, dorsal face of petiole slightly convex without distinct edge, PEW/CS <0.156 (Fig. 8B); propodeum more rectangular, with a raised anterodorsal transverse carina; integument of mesonotum and propodeum strongly sculptured (Fig. 8B); antennal funiculus, basitarsi and tarsi brown	***jodina***

**Figure 4.**
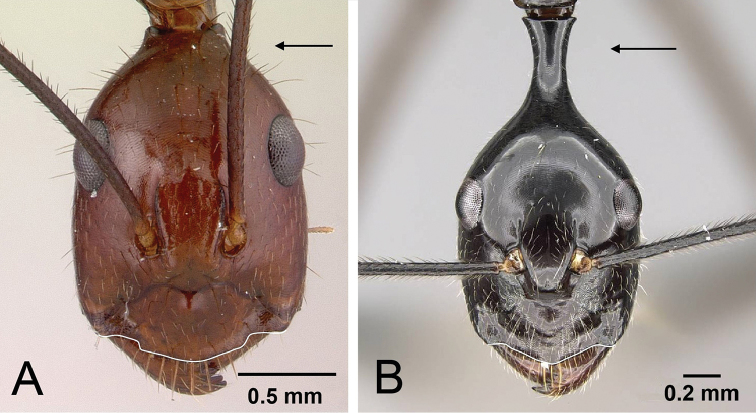
Illustration of the neck (posterior region of the head) and the anterior clypeal margin of **A** Camponotus imitator (CASENT0452849), and **B** *Camponotus karaha* (CASENT0152090). Arrows indicate the posterior portion of the head capsule which in *karaha* is drawn out into a strongly constricted neck, behind which the head capsule flares out, forming a pronounced collar.

**Figure 5.**
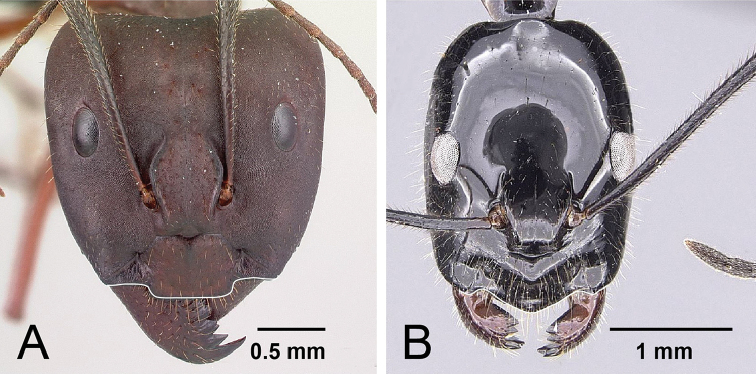
Head of major worker in full-face view. **A** *Camponotus imitator* (CASENT0452863), head wedge-shaped **B** *Camponotus karaha* (CASENT0151921), head rectangular.

**Figure 6.**
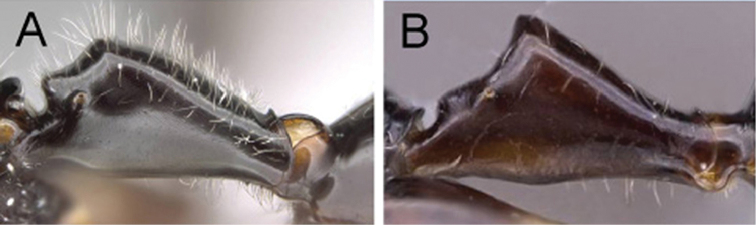
Petiole in lateral view of **A** *Camponotus longicollis* (CASENT0191989), and **B** *Camponotus karaha* (CASENT0067555).

**Figure 7.**
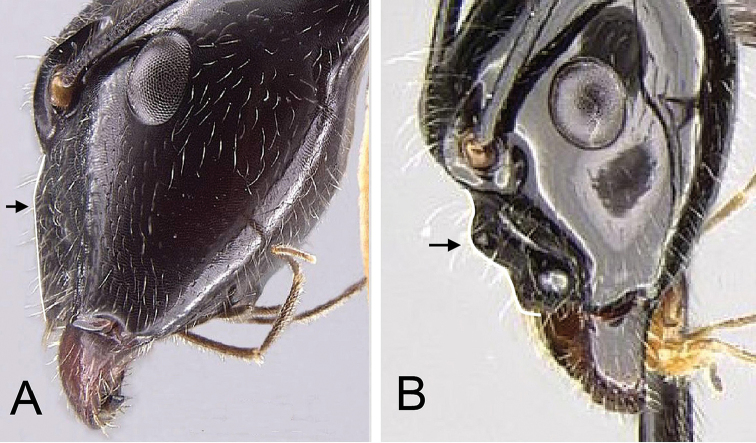
Head in lateral view showing the dorsal margin of the clypeus (similar in minor and major workers). **A** *Camponotus longicollis* (CASENT0191989) **B** *Camponotus karaha* (CASENT0353274).

**Figure 8.**
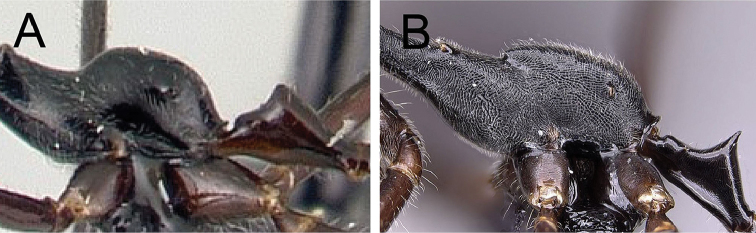
Lateral view of propodeum and petiole in minor workers of **A** *Camponotus karaha* (CASENT0067555), and **B** *Camponotus jodina* (CASENT0152090).

#### Camponotus imitator

Taxon classificationAnimaliaHymenopteraFormicidae

Forel, 1891

**Figure 9.**
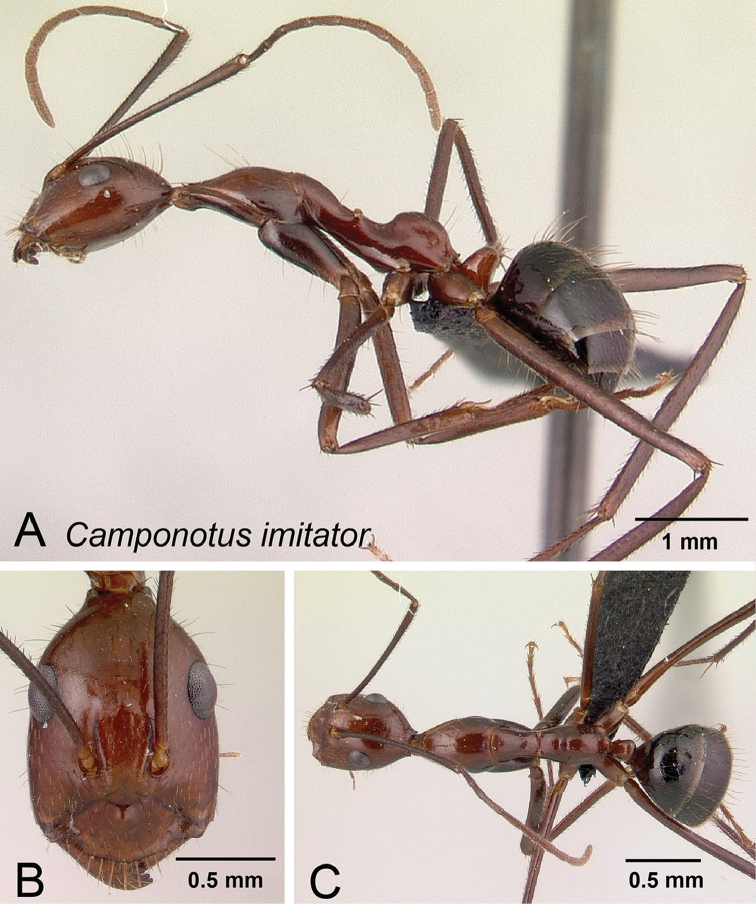
*Camponotus imitator* minor worker CASENT0452849. **A** Lateral view **B** Head in full-face view **C** Dorsal view.

**Figure 10.**
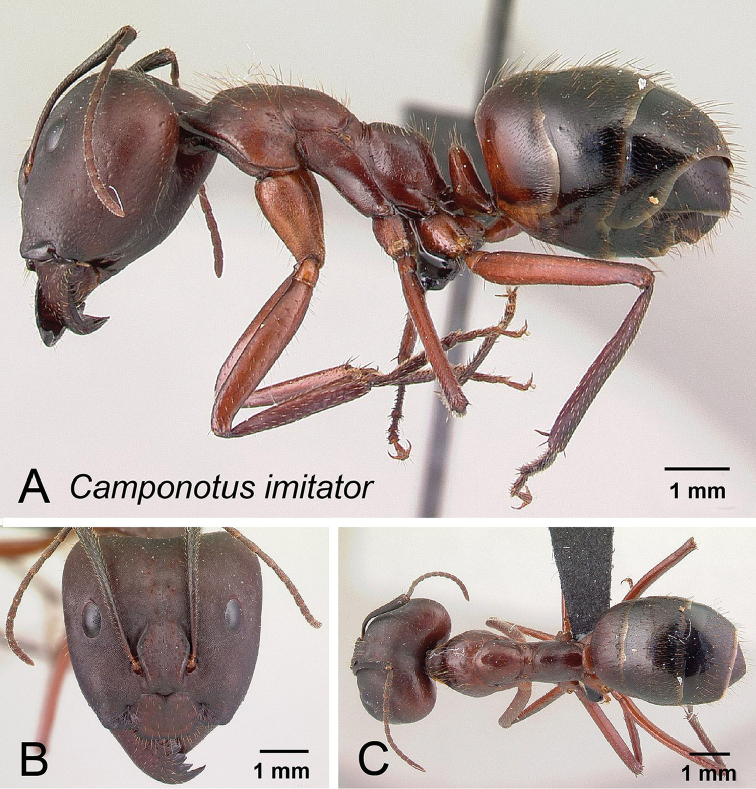
*Camponotus imitator* major worker CASENT0452863. **A** Lateral view **B** Head in full-face view **C** Dorsal view.

**Figure 11.**
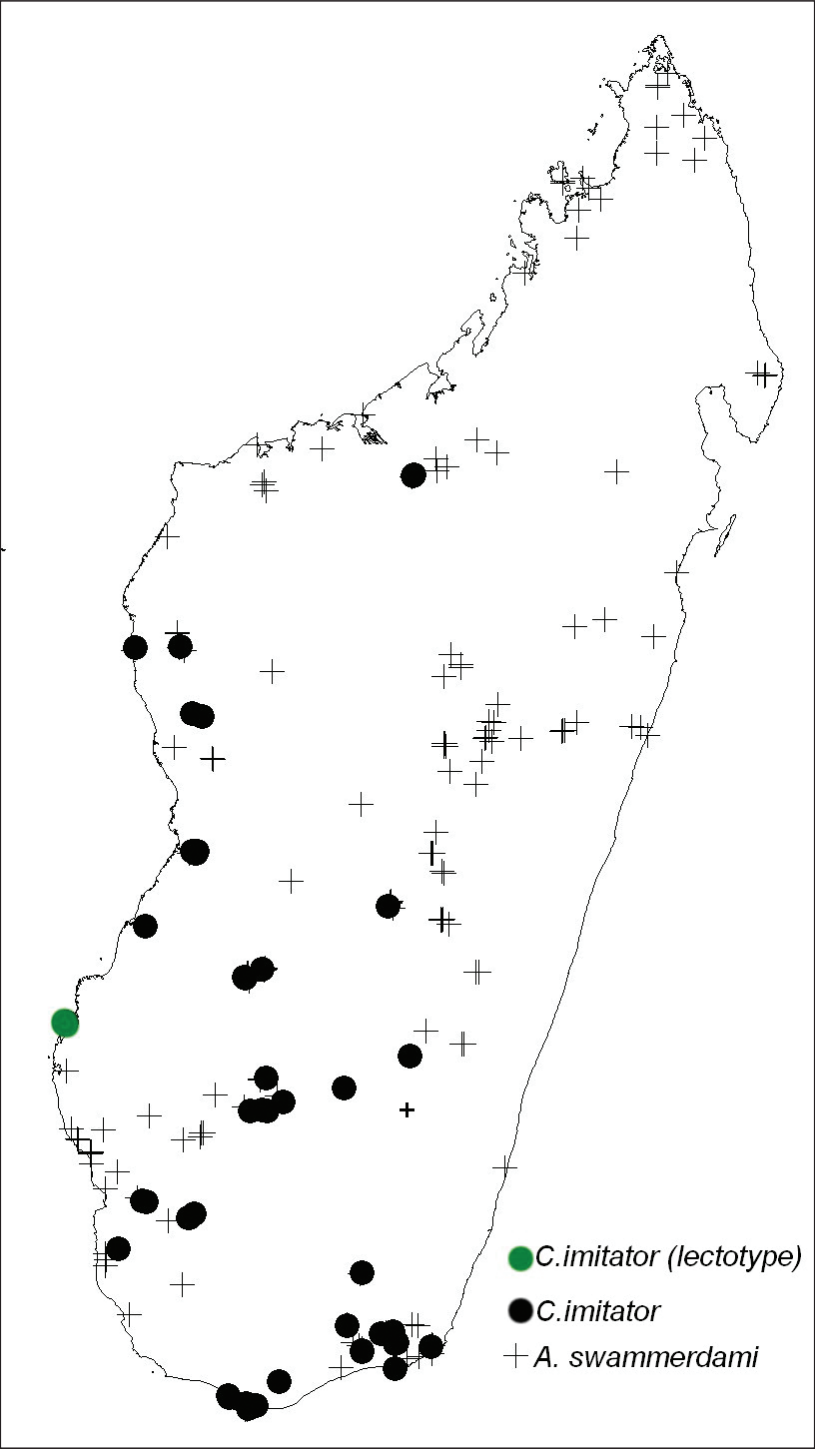
*Camponotus imitator* is sympatric with *A. swammerdami* through most of its range.

Figures 1B , 4A , 5A , 9 , 10 , 11

Camponotus imitator Forel, 1891: 209, pl. 4, fig. 15; pl. 5, fig. 8. Lectotype worker, present designation, MADAGASCAR, Province Toliara, Morandava [-20.2833, 44.28333] (coordinates obtained from MBG Gazetteer), (Grevé), CASENT0101365 (MHNG) [not examined morphometrically] and two paralectotypes, workers, MADAGASCAR, CASENT0101116 (NHMB): CASENT0104647 (ZMHB) [morphometrically not examined]. [Combination in Camponotus (Myrmosphincta): Forel, 1912: 92; in Camponotus (Myrmopytia): Emery, 1920: 257].
Camponotus imitator var. resinicola Santschi, 1911 133. Lectotype worker, present designation, MADAGASCAR, Ambolisatra (probably today’s Ambolisaka), [-21.7333, 43.36666], 6-7-1898, (C. Grandidier), CASENT0101117 (NHMB) [examined] and one paralectotype worker, Region du Sud, Andrahomana, [-25.183333, 46.63333], Nov. 1901 (Ch. Alluaud), CASENT0101118 (NHMB), and one worker Region du Sud-Est, Fort-Dauphin, [-25.03333, 46.98333], Août, 1901 (Ch. Alluaud), CASENT0101119 (NHMB) [not examined morphometrically]. **syn. n.** [Raised to species: Wheeler, W.M. 1922: 1049. Reverted to subspecies of imitator: Emery, 1925: 115].

##### Diagnosis.

*Camponotus imitator* is easily recognizable within the group on the basis of the following combination of characters: posterior region of head only slightly extended, not narrowed into a long neck in the minor worker, PoOC/CL 0.256 [0.224, 0.304] and trapezoidal in major worker, CWb/CL 0.951 [0.841, 1.031]; anterior clypeal margin with a rectangular projection ClyL/CL 0.289 [0.231, 0.316]; petiole nodiform PEW/CS 0.224 [0.203, 0.255]; both castes bicolored: head, mesosoma and appendages reddish brown to dark brown, gaster black (minor) to mainly black (major).

##### Description of minor worker.

Head suboval, posterior region of head only slightly and broadly extended with margins weakly convex. (CS) 1.68 mm [1.34, 2.18] (n=25). Standing setae present on posterolateral margin of head and vertex in full-face view. Eyes situated on posterior half of head, PoOC/CL 0.253 [0.224, 0.293]. Frontal carina convex, FR/CS 0.254 [0.209, 0.280], antennal scape surpassing posterior margin of head by more than half its length, SL/CS 1.517 [1.211, 1.731]. Anteromargin of clypeus with broad rectangular projection, posterior margin concave, ClyL/CL 0.271 [0.231, 0.293]; mandible with six teeth, palps long with respect to head size.

Pronotum weakly undulant. Suberect pronotal setae numerous (more than 12). Mesonotum straight, MPD/CS 1.181 [0.954, 1.403]. Erect mesonotal setae varying from absent to numerous (two or three pairs anterior to mesothoracic spiracle). Mesothoracic spiracles prominent; propodeal dorsum protuberant. Erect propodeal setae moderate in number (4–6). ML/CS 1.957 [1.670, 2.149]. Petiole nodiform, dorsum of node convex, petiole higher than broad, PEW/CS 0.242 [0.164, 0.264]. Erect setae present on petiolar apex.

Color: head and mesosoma red to reddish brown, gaster dark brown to black. Erect setae light brown. Sparse appressed pubescence present.

##### Description of major worker.

In full-face view, head truncated posteriorly, evenly tapering to base of mandibles, posterior margin of head weakly concave. Absolute cephalic size (CS) 3.26 mm [2.32, 3.94] (n=22). Cephalic margin with scattered short hairs; cephalic dorsum coarsely reticulate-foveolate. Eye situated on posterior half of the head, PoOC/CL 0.284 [0.242, 0.323]. Frontal carinae sinuate, FR/CS 0.260 [0.209, 0.280], coronal line distinct, antennal scape just surpassing the posterior margin of head by length of one funiculus segment, SL/CS 0.853 [0.711, 1.155]. Anterior margin of clypeus with a rectangular projection, medially straight to slightly convex, ClyL/CL 0.294 [0.269, 0.326]; masticatory margin of mandible with 7–10 teeth, microreticulate at base, becoming finely striolate apically, with scattered piligerous punctures, rarely with a few weak longitudinal rugae near base.

Dorsal outline of mesosoma complex. Promesonotum forms a regular convexity with a shallow impression at the promesonotal suture and is stepped to the propodeal dorsum. Suberect promesonotal setae inclined anteriorly, ML/CS 1.337 [1.168, 1.655]; metanotum distinct; propodeal dorsum almost straight to evenly convex, posterodorsal margin forms rounded angle with declivity.

Petiole higher than broad, node summit flat; brown standing setae present on entire dorsum. PEW/CS 0.221 [0.199, 0.247].

Color: head, mesosoma, petiole, and base of first gastral segment reddish brown, remainder of gaster dark brown to black. Scattered appressed pubescence generally present. Setae light brown.

##### Distribution and biology.

The minor worker of *Camponotus imitator* is thought to mimic the myrmicine ant *Aphaenogaster swammerdami* due to its color and the form of its constricted mesonotum and shape of propodeum, which could appear as a petiole in dorsal view (Forel 1891) (Fig. 11). This myrmicine nests underground and shares its nests with snakes, *Madagascarophis colubrinus* (Schlegel, 1837) and *Leioheterodon modestus* (Günther, 1863); it is an important secondary seed disperser of *Commiphora guillaumini* (Burseraceae) (Böhning-Gaese et al. 1999).

*Camponotus imitator* is distributed in the dry forest and woodland of western and southern Madagascar at elevations ranging from 25 m to 990 m (Fig. 11). Its distribution is sympatric with *A. swammerdami* through most of its range (Fig. 11). It has been collected by litter sifting, Malaise and pitfall traps, as well as beating low vegetation and from the ground in rotten logs. This species nests underground.

##### Comment.

We propose that *Camponotus imitator resinicola* (Santschi, 1911) is synonymized with *Camponotus imitator* Forel. In the original descriptions, the former differs from the latter by the presence of reddish patches on the first gastral segment near the petiolar insertion. Examination of material from 10 collection events of *C. imitator* colonies indicates that this trait is highly variable within colonies, and no other reliable characters were found to separate the subspecies from *imitator*. Moreover, no other qualitative trait or biogeographic evidence exists that would underpin the subspecies status of *resinicola*.

##### Additional material examined.

Province **Fianarantsoa**: Tsaranoro, 32.8 km 230° Ambalavao, -22.08317, 46.774, 975 m, savannah woodland (B.L. Fisher et al.) (CASC); Parc National d’Isalo, Ambovo Springs, 29.3 km 4° N Ranohira, -22.29833, 45.35167, 990 m, Uapaca woodland (Fisher, Griswold et al.) (CASC); Ihosy, -22.40317, 46.12917, 735 m, urban/garden (B.L.Fisher et al.) (CASC); Forêt d’Atsirakambiaty, 7.6 km 285° WNW Itremo, -20. 59333, 46.56333, 1550 m, grassland (Fisher, Griswold et al.) (CASC). Province **Mahajanga**: Boeny Region,Distric of Marovoay, Ampijoroa National Park, 160 km North of Maevatanana on RN 04, -16.31933, 46.81333, 42 m, Decidious forest (Rinha, Mike) (CASC); Réserve forestière Beanka, 50.2 km E Maintirano, -18.02649, 44.05051, 250m, tropical dry forest on tsingy (B.L.Fisher et al.) (CASC); Station Forestiere Ampijoroa, -16.31667, 46.81667, 80 m, tropical dry forest (P.S.Ward) (PSWC); Antsalova, -18.68333, 44.61667, 100 m (D. Lees) (PSWC); Parc National Tsingy de Bemaraha, 10.6 km ESE 123° Antsalova, -18.70944, 44.71817, 150 m, tropical dry forest on Tsingy (Fisher-Griswold Arthropod Team) (CASC). Province **Toliara**: 45km NE Morondava, -20.05, 44.61667, 30 m, tropical dry forest (P.S.Ward) (PSWC); 48km ENE Morondava, -20.06667,44.65,30 m, tropical dry forest (D.M.Olson) (PSWC); Sept Lacs, -23.52472, 44.15917, 160 m, Spiny thicket Gallery forest transition (Frontier Project) (CASC); Forêt de Kirindy, 15.5 km 64° ENE Marofandilia, -20.045, 44.66222, 100 m, tropical dry forest (B.L.Fisher et al.) (CASC); Parc National de Tsimanampetsotsa, Forêt de Bemanateza, 20.7 km 81° E Efoetse, 23.0 km 131° SE Beheloka, -23.99222, 43.88067, 90 m, spiny forest/thicket (Fisher-Griswold Arthropod Team) (CASC); Parc National d’Andohahela, Forêt de Manatalinjo, 33.6 km 63° ENE Amboasary, 7.6 km 99° E Hazofotsy, -24.81694, 46.61, 150 m, spiny forest/thicket (Fisher-Griswold Arthropod Team) (CASC), Parc National d’Andohahela, Forêt de Manatalinjo, -24.82466, 46.60111, 100 m,spiny forest/thicket (Fisher-Griswold Arthropod Team) (CASC); Parc National de Kirindy Mite, 16.3 km 127° SE Belo sur Mer, -20.79528, 44.147, 80 m, tropical dry forest (Fisher-Griswold Arthropod Team) (CASC); Parc National d’Andohahela, Forêt d’Ambohibory, 1.7 km 61° ENE Tsimelahy, 36.1 km 308° NW Tolagnaro, -24.93, 46.6455, 300 m, tropical dry forest (Fisher-Griswold Arthropod Team) (CASC); Forêt de Kirindy, 15.5 km 64° ENE Marofandilia,-20.045,44.66222,100 m, tropical dry forest (Fisher-Griswold Arthropod Team) (CASC); Anosy Region, Distric of Amboasary, 58Km SW of Fort Dauphin, 08 Km NW of Amboasary, Berenty Special Reserve, -25.00667, 46.30333, 85 m, Galery forest (Rin’ha, Mike) (CASC); Tsihombe, -25.31833, 45.48367, 30 m, urban/garden (B.L.Fisher et al.) (CASC); Forêt Vohidava 89.6 km N Amboasary, -24.23333, 46.30167, 230 m, spiny forest/thicket (B.L.Fisher et al.) (CASC); Forêt de Kirindy, 15.5 km 64° ENE Marofandilia,-20.06855,44.65956667,30 m, tropical dry forest (B.L.Fisher) (CASC); Anosy Region, Distric of Fort-Dauphin, Andohaela National Park Parcelle II, Tsimela,42Km W of Fort-Dauphin, -24.93683, 46.62667, 177 m, transition forest (Michael Irwin, Frank Parker, Rin’ha) (CASC); Forêt de Kirindy, 15.5 km 64° ENE Marofandilia, -20.06915, 44.66041667,30 m, tropical dry forest (B.L.Fisher) (CASC); Atsimo Andrefana Region, Distric of Betioky ; Beza Mahafaly Special reserve Parcelle Belle vue 07 Km W of Research Station, -23.68983, 44.5755, 177 m, spiny forest (Rin’ha) (CASC); Réserve Berenty,-25.01667,46.3,25 m,tropical dry forest (P.S.Ward) (PSWC); Res. Beza-Mahafaly, Parcel 1, -23.65, 44.63333, 130 m, tropical dry forest (P.S.Ward) (PSWC); Makay Mts., -21.31334, 45.14525,575 m, Burned savannah (B.L.Fisher et al.) (CASC). Makay Mts., -21.29961, 45.12919, 570 m, Dry forest edge and burned savannah (B.L.Fisher et al.) (CASC); Makay Mts., -21.22344, 45.3135, 550 m, Gallery forest with bamboo (B.L.Fisher et al.) (CASC); Forêt de Mahavelo, Isantoria River,-24.75833,46.15717,110 m,spiny forest/thicket (Fisher-Griswold Arthropod Team) (CASC); 7.0 km 156° SSE Lavanono,-25.47111,44.9885,50 m,spiny forest/thicket (Fisher-Griswold Arthropod Team) (CASC); 4.4 km 148° SSE Lavanono, -25.45056, 44.97417, 60 m, spiny forest/thicket (Fisher-Griswold Arthropod Team) (CASC); 12.7 km 287° W Marovato,-25.53611,45.15017,130 m, spiny forest/thicket (Fisher-Griswold Arthropod Team) (CASC); 3.5 km 236° SW Marovato, -25.55389, 45.25583, 230 m, spiny forest/thicket (Fisher-Griswold Arthropod Team) (CASC); Réserve Spéciale de Cap Sainte Marie, 12.3 km 262° W Marovato,-25.58167,45.16833,200 m, spiny forest/thicket (Fisher-Griswold Arthropod Team) (CASC); Forêt de Mite, 20.7 km 29° WNW Tongobory, -23.52417, 44.12133, 75 m, gallery forest (Fisher-Griswold Arthropod Team) (CASC); Tsimelahy - Parcel II, Andohahela National Park, transition forest, Tulear Province, -24.93683, 46.62667, 180 m, transition forest (M.E. Irwin, F.D. Parker, R. Harin’Hala) (CASC); Andohaela N. P., Tsimelahy, -24.93683, 46.62667, 180 m, transition forest (M.E. Irwin, F.D. Parker, R. Harin’Hala) (CASC); Ihazofotsy - Parcel III, Andohahela National Park, transition forest, Tulear Province, -24.83483, 46.48683, 80 m, transition between spiny and dry deciduous forests (M.E. Irwin, F.D. Parker, R. Harin’Hala) (CASC);Parcel I, Beza Mahafaly Reserve, near research station, Tulear Province, -23.6865, 44.591, 165 m, dry deciduous forest (R. Harin’Hala) (CASC); Parcel II, Beza Mahafaly Reserve, near Bellevue, Tulear Province, -23.68983, 44.5755, 180 m, spiny forest (R. Harin’Hala) (CASC).

#### Camponotus jodina sp. n.

Taxon classificationAnimaliaHymenopteraFormicidae

http://zoobank.org/FFE73B10-0111-40F5-831B-AC72F3D89E0E

**Figure 12.**
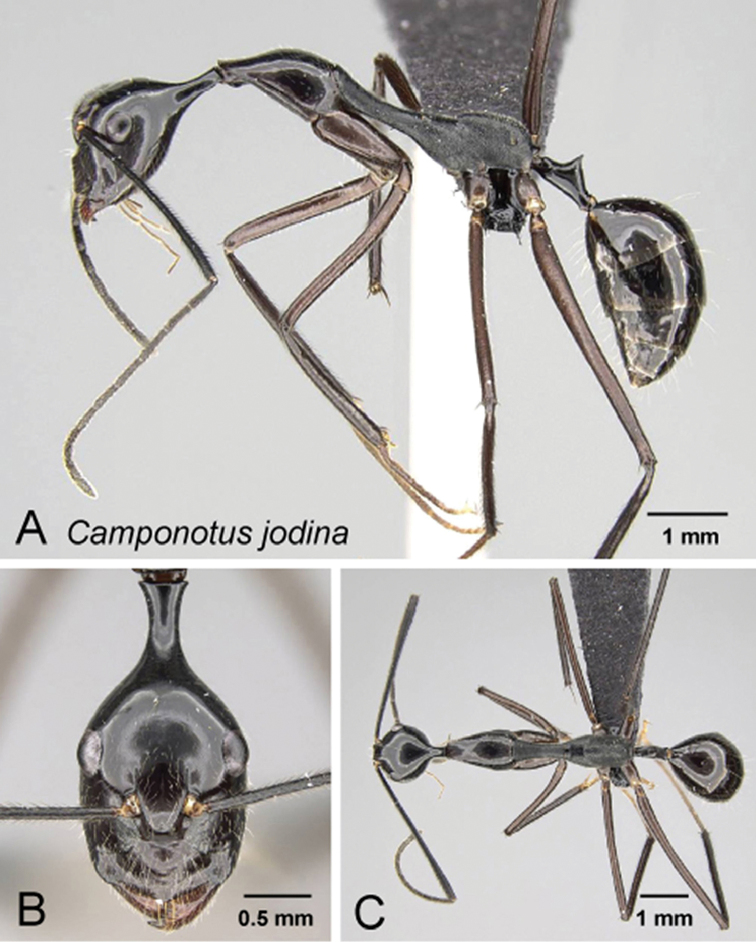
*Camponotus jodina* minor worker CASENT0152090. **A** Lateral view **B** Head in full-face view **C** Dorsal view.

Figures 8B , 12 , 18

##### Type material investigated.

Holotype worker: MADAGASCAR, Province Toamasina, Parc National de Zahamena, Onibe River, -17.75908 48.85468, 780 m, 22.ii.2009, rainforest, on low vegetation. (B.L. Fisher et al.). Collection code: BLF22344. Unique specimen identifier: CASENT0152090 (CASC).

Paratype worker: MADAGASCAR, Prov. Toamasina, Parc National de Zahamena, Sahavorondrano River, -17.75257, 48.85725, 765 m, 23.ii.2009, rainforest, beating low vegetation. (B.L. Fisher et al.). Collection code: BLF22401. Unique specimen identifier: CASENT0153052 (CASC).

##### Diagnosis.

*Camponotus jodina* is easily distinguishable from the other species of *Myrmopytia* on the basis of the following character combination: petiole surmounted by a conical node terminated with a blunt spine, PEW/CS 0.152 [0.149, 0.156], propodeum coarsely reticulate with a short vertical anterior face.

##### Description of minor worker.

Head longer than wide, rear portion of head extended into a long neck, sides of head narrowed in front of eyes. CS 1.84 mm [1.80, 1.88] (n=2). Posterior margin of head glabrous, suberect genal setae present on sides of head in full-face view. Eyes located anterior to the midlength of head capsule in full-face view, PoOC/CL 0.417 [0.407, 0.426]. Frontal carina convex, FR/CS 0.228 [0.222, 0.234]. Scape distinctly surpassing posterior margin by almost half of its length, SL/CS 1.870 [1.851, 1.889]. Anterior margin of clypeus produced into an obtuse angle, lateral border straight. In profile, posteromedian portion of clypeus with an obtuse angle. ClyL/CL 0.225 [0.213, 0.237]. Mandible with 6 teeth, palps long with respect to head size.

Pronotum evenly curved over its length. Mesonotum straight and elongate, MPD/CS 1.424 [1.422, 1.426]. Erect setae absent, appressed pubescence generally sparse. Mesothoracic spiracles prominent; propodeal dorsum evenly convex, its outline meeting the metanotal groove with transverse carina. ML/CS 2.294[2.255, 2.333].

Petiole nodiform. Dorsum of petiole strongly concave, tapering apically to a blunt spine, PEW/CS 0.152 [0.149, 0.156].

Entire body including antennae dark brown. Coxae, femora, and tibiae brown. Head, pronotum, fore-coxae and petiole microreticulate, mesonotum and propodeum reticulate-costate.

##### Major worker.

Unknown.

##### Queen.

Unknown.

##### Male.

Unknown.

##### Distribution and biology.

*C. jodina* is known from only two specimens collected in Parc National de Zahamena, in rainforest, at two different localities: Onibe River and Sahavorondrano River (Fig. 18A). Workers were found foraging on lower vegetation. *C. jodina* occurs sympatric with *C. karaha* at the Sahavorondrano River.

##### Etymology.

This species is named for the shape of its petiole in profile; the Malagasy word “jodina” means directed upward.

#### Camponotus karaha sp. n.

Taxon classificationAnimaliaHymenopteraFormicidae

http://zoobank.org/35B73CB2-30F5-4EAF-8321-F93DFADD95EB

Figures 4B , 5B , 6B , 7B , 8A , 13 , 14 , 15 , 18

##### Type material investigated.

Holotype worker: MADAGASCAR, Province Antsiranana, Parc National de Marojejy, Manantenina River, 27.6 km 35° NE Andapa, 9.6 km 327° NNW Manantenina, -14.435 49.76, 16.xi.2003, 775 m, rainforest, ex rotten log. (B.L. Fisher et al.). Collection code: BLF08983 Unique specimen identifier: CASENT0487715 (CASC).

Paratype workers: Six workers with the same data as holotype, Collection code: BLF08983, Unique specimen identifiers: CASENT0487711 (2w), CASENT0487712 (2w), CASENT0487713 (2w), (CASC).

##### Diagnosis.

Workers of *Camponotus karaha* can be differentiated from the other three species by the triangular form of the petiole in lateral view, and the protruding clypeus.

**Figure 13.**
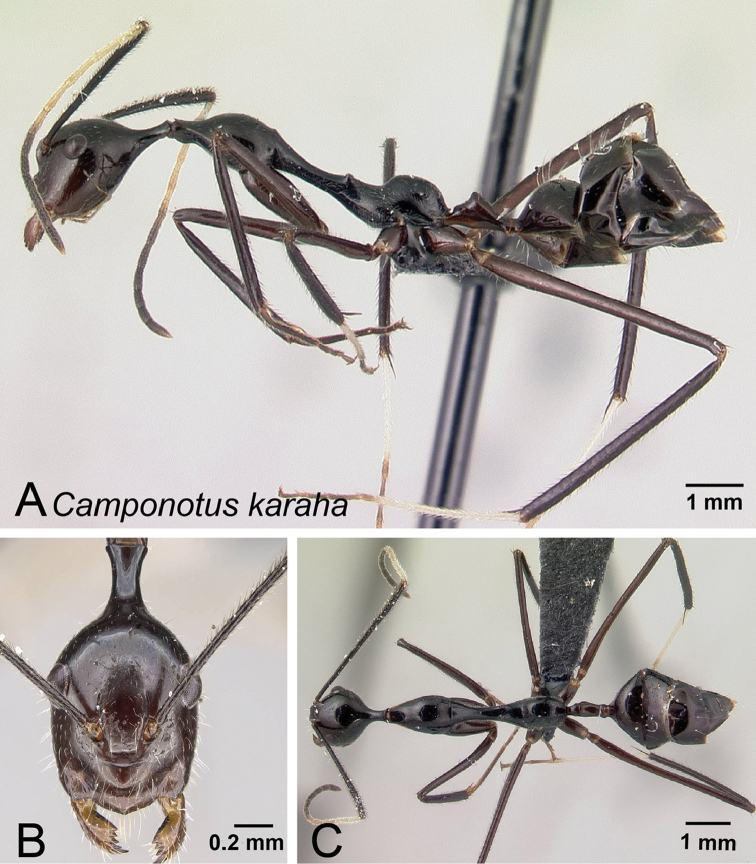
*Camponotus karaha* minor worker CASENT0067555. **A** Lateral view **B** Head in full-face view **C** Dorsal view.

**Figure 14.**
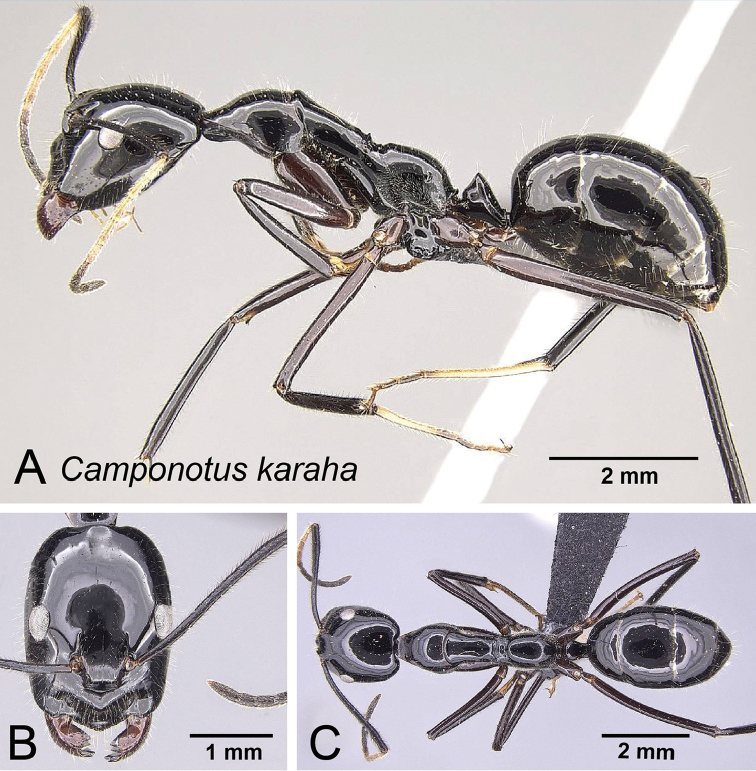
*Camponotus karaha* major worker CASENT0151921. **A** Lateral view **B** Head in full-face view **C** Dorsal view.

**Figure 15.**
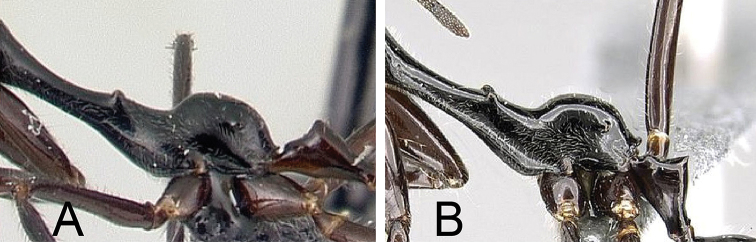
**A** Variant 1, a typical *C. karaha*. CASENT0067555 **B** Variant 2 from the north. CASENT0353274.

##### Description of minor worker.

Head longer than wide, anterior region of head elongated, caudate, sides of the head narrowed in front of eyes, CS 1.723 mm [1.35, 1.92] (n=47). Erect filiform setae on lateral margin of head and on gena. Eyes located anterior to the midlength of sides of head in full-face view, PoOC/CL 0.431 [0.404, 0.473]. Frontal carinae convex, FR/CS 0.238 [0.213, 0.267]. Scape distinctly surpassing posterior margin by almost half of its length, SL/CS 1.832 [1.651, 2.051]. Anterior margin of clypeus produced into an obtuse angle; in lateral view, clypeus protruding strongly, anterior portion may appear indented (Fig. 7B), masticatory margin with 6 teeth, palps long with respect to head size.

Pronotum weakly undulant. Mesonotum straight and elongate, distinctly compressed laterally anterior to mesothoracic spiracles, MPD/CS 1.401 [1.196, 1.595]. Erect setae absent, appressed pubescence sparsely distributed. Mesothoracic spiracles prominent; propodeal dorsum protuberant, noticeably convex. ML/CS 2.203 [1.989, 2.405].

Petiole conical, petiolar apex with an obtuse angle, posterior face of petiole flat to slightly convex, sides always distinct. PEW/CS 0.198 [0.169, 0.244].

Entire body generally black, pronotum may vary to brown, coxae a lighter color than mesosoma, basitarsus and up to 5 basal funiculi whitish. Mesonotum and propodeum smooth and glabrous to microsculptured with sparse appressed to subdecumbent setae.

##### Description of major worker.

Head subrectangular in full face view, occipital portion broad, sides of the head mostly parallel and weakly convex, CS 2.108 mm [1.92, 2.28] (n=9). Erect filiform setae present on entire head capsule. Eyes located at midlength of head capsule in full-face view. PrOc/CL 0.434 [0.417, 0.455]. Frontal carinae straight posterior to antennal insertion and curving smoothly toward the posterior margin of clypeus. FR/CS0.270 [0.255, 0.292]. Scape surpassing posterior margin by one fourth of its length. SL/ CS 0.971 [0.711, 1.331]. Anterior margin of clypeus projecting to an obtuse angle and, in lateral view, posterior portion of clypeus weakly produced dorsally (Fig. 7B) masticatory margin of mandible with 6 teeth, palps long with respect to head size.

Pronotum weakly undulant. Anterior region of mesonotum (immediately posterior to the pro-mesonotal suture) in profile rising above pronotum. Dorsal outline of mesonotum and propodeum form a continuous straight line interrupted by a shallow metanotal groove. Erect setae present, appressed pubescence sparsely distributed. Mesothoracic spiracle feebly produced laterally. ML/CS 1.872 [1.800, 2.009].

In profile, petiole conical, node summit acute. PEW/CS 0.222 [0.211, 0.250].

Entire body black; femora and tibia dark brown, basitarsus of second and third legs light brown, funicular segments light brown, becoming dark apically. Pronotum and mesonotum microreticulate, sides of propodeum finely punctate, standing filiform setae present in all surfaces.

##### Distribution and biology.

*Camponotus karaha* is currently known from 14 localities along the eastern rainforest and montane rainforest of Madagascar at elevations ranging from 175 to 1325 m (Fig. 18B). Specimens have been collected on the ground in rotten logs and on low vegetation.

##### Etymology.

The Malagasy word “karaha” means similar, look-alike.

##### Notes on morphological variability.

Workers of *C. karaha* exhibit morphological variability in qualitative traits such as sculpture, color of mesosoma, and profile of propodeum that differ between populations. This divergence is not, or barely, supported by multivariate analyses involving 19 quantitative traits. For this reason, we conclude that all populations examined represent a single species and ascribe the variation to intraspecific variability of populations occupying diverse sites, making geographic (e.g. elevation) or ecological factors as possible explanations for the variance. Populations from the north of its distribution range differ notably in shape of propodeal dorsum and petiolar node and further research on additional samples are needed to further evaluate species status of these populations. For now, we note the differences of the northern populations from the more typical *karaha*.

**Figure 16.**
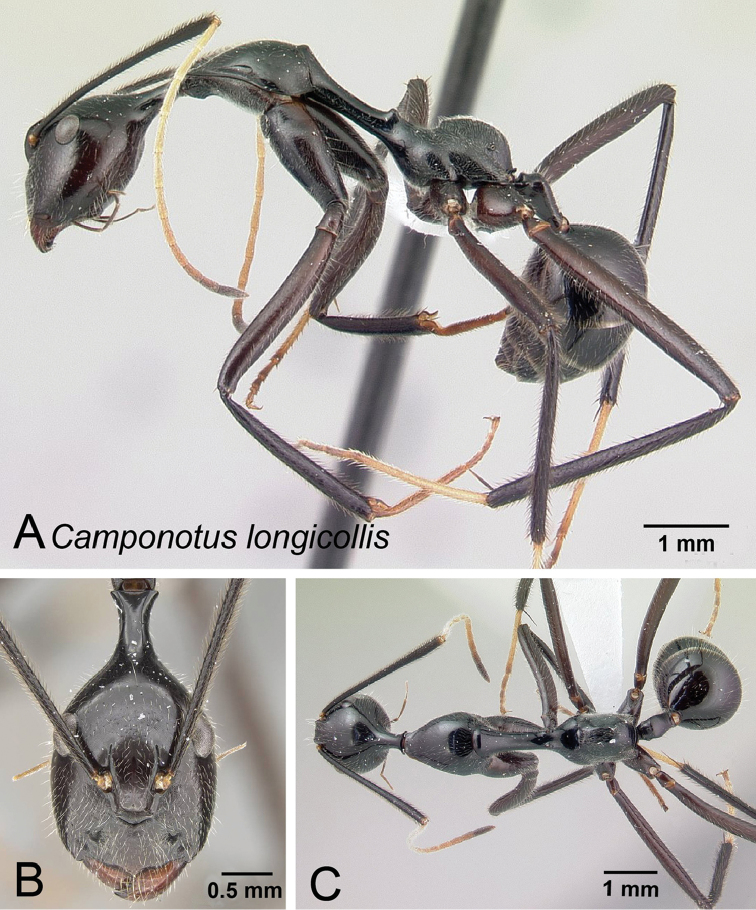
*Camponotus longicollis* minor worker CASENT0191989. **A** Lateral view **B** Head in full-face view **C** Dorsal view.

**Variant 1.** This variant is the typical *C. karaha*, and is fairly widespread throughout the eastern rainforest of Madagascar. It can be recognized by having a propodeum dorsum smoothly convex in lateral view, declivitous face of petiole distinctly flat with defined lateral margins, petiolar apex forms a right angle (Fig. 16A).

**Variant 2.** Workers are known from montane rainforest of Montagne d’Anjanaharibe and Parc National Masoala, Ambanizana, elevation 800–1100 m. It can be differentiated from the other morph by the propodeal dorsum almost flat in lateral view, declivitous face of petiole convex and not marginate laterally, petiolar apex acuminate (Fig. 16B).

##### Additional material examined.

Province **Antsiranana**: 6.5 km SSW Befingotra, Rés. Anjanaharibe-Sud, -14.75, 49.5, 875 m (B.L.Fisher) (CASC); 9.2 km WSW Befingotra, Rés. Anjanaharibe-Sud, -14.75, 49.46667, 1180 m (B.L.Fisher) (CASC); Betaolana Forest, along Bekona River, -14.52996, 49.44039, 880 m (B.L.Fisher et al.) (CASC); Betaolana forest, Ambodihazovolabe village along Ambolokopatrika river, -14.54484, 49.45163, 740 m (B.L.Fisher et al.) (CASC); Binara Forest, -13.26392, 49.59919, 1065 m (B.L.Fisher et al.) (CASC); Binara Forest, -13.26388, 49.60141, 900 m (B.L.Fisher et al.) (CASC); Galoko chain, Mont Kalabenono, -13.63999, 48.67374, 498 m (B.L.Fisher et al.) (CASC); Galoko chain, Mont Kalabenono, -13.64179, 48.67282, 643 m (B.L.Fisher et al.) (CASC); Galoko chain, Mont Kalabenono, -13.64609, 48.67732, 937 m (B.L.Fisher et al.) (CASC); Makirovana forest, -14.17066, 49.95409, 415 m (B.L.Fisher et al.) (CASC); Makirovana forest, -14.16044, 49.95216, 550 m (B.L.Fisher et al.) (CASC); Makirovana forest, -14.16666, 49.95, 715 m (B.L.Fisher et al.) (CASC); Makirovana forest, -14.16506, 49.9477, 900 m (B.L.Fisher et al.) (CASC); Parc National de Marojejy, Antranohofa, 26.6 km 31° NNE Andapa, 10.7 km 318° NW Manantenina, -14.44333, 49.74333, 1325 m (B.L.Fisher) (CASC); Parc National de Marojejy, Manantenina River, 27.6 km 35° NE Andapa, 9.6 km 327° NNW Manantenina, -14.435, 49.76, 775 m (B.L.Fisher et al.) (CASC); Parc National de Marojejy, Manantenina River, 28.0 km 38° NE Andapa, 8.2 km 333° NNW Manantenina, -14.43667, 49.775, 450 m (B.L.Fisher et al.) (CASC); RNI Marojejy, 10km NW Manantenina, -14.43333, 49.76667, 750 m (E.L. Quinter) (CASC). Province **Fianarantsoa**: 7.6 km 122º Kianjavato, Forêt Classée Vatovavy, -21.4, 47.94, 175 m (B.L.Fisher et al.) (CASC) ; Foret d’Ambalagoavy Nord, Ikongo, Ambatombe, -21.8275, 47.33889, 625 m (R. Harin’Hala & M.E. Irwin) (CASC); Forêt de Vevembe, 66.6 km 293° Farafangana, -22.791, 47.18183, 600 m (B.L. Fisher et al.) (CASC). Province **Toamasina**: 6.9 km NE Ambanizana, Ambohitsitondroina, -13.56667, 50, 1080 m (B.L.Fisher) (CASC); Ambanizana, Parc National Masoala, -15.57167, 50.00611, 800–897 m (D. Andriamalala, D. Silva, et al.) (CASC); Ankerana, -18.4017, 48.80605, 1035 m (B.L.Fisher et al.) (CASC); Ankerana, -18.4061, 48.82029, 725 m (B.L.Fisher et al.) (CASC); Ankerana, -18.40062, 48.81311, 865 m (B.L.Fisher et al.) (CASC); Montagne d’Anjanaharibe, 18.0 km 21° NNE Ambinanitelo, -15.18833, 49.615, 470 m (Fisher, Griswold et al.) (CASC); Montagne d’Anjanaharibe, 19.5 km 27° NNE Ambinanitelo, -15.17833, 49.635, 1100 m (Fisher, Griswold et al.) (CASC); Parc National de Zahamena, Besaky River, -17.75244, 48.85321, 760 m (B.L.Fisher et al.) (CASC); Parc National de Zahamena, Sahavorondrano River, -17.75257, 48.85725, 765 m (B.L.Fisher et al.) (CASC); Parc National Mananara-Nord, 7.1 km 261° Antanambe, -16.455, 49.7875, 225 m (B.L.Fisher et al.) (CASC); Réserve Nationale Intégrale Betampona, Betampona 35.1 km NW Toamasina, -17.91801, 49.20074, 500 m (B.L.Fisher et al.) (CASC); Réserve Naturelle Betampona, 34.1 km 332° Toamasina, -17.916135, 49.20185, 550 m (B.L.Fisher) (CASC).

#### Camponotus longicollis sp. n.

Taxon classificationAnimaliaHymenopteraFormicidae

http://zoobank.org/61EB2C24-CB44-4B54-B98F-AEE4CCE18ABC

**Figure 17.**
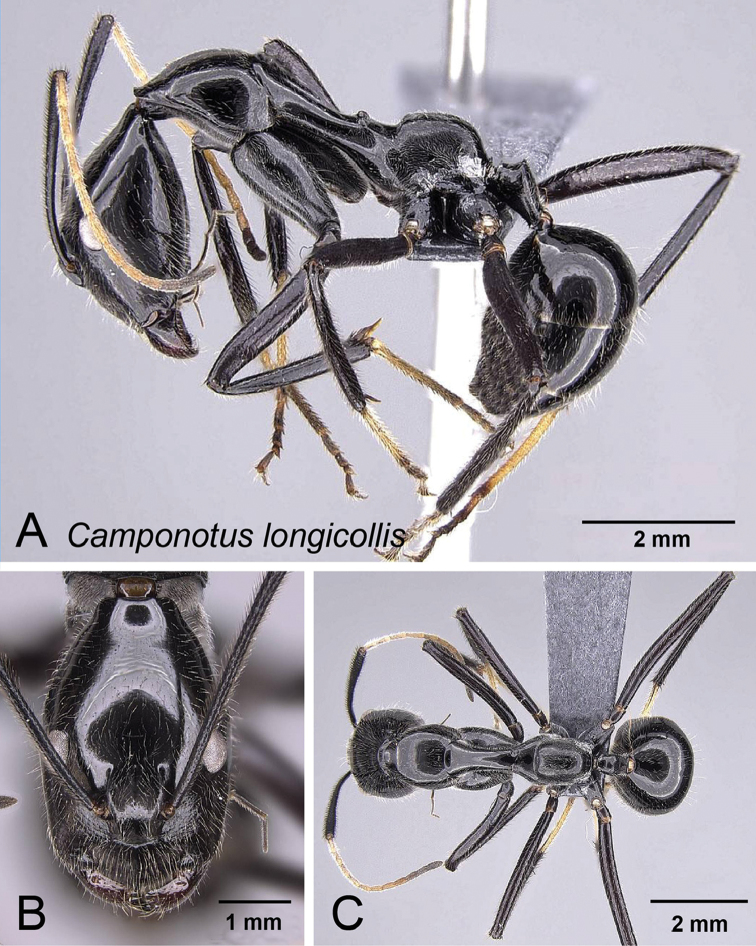
*Camponotus longicollis* major worker CASENT0763008. **A** Lateral view **B** Head in full-face view **C** Dorsal view.

**Figure 18.**
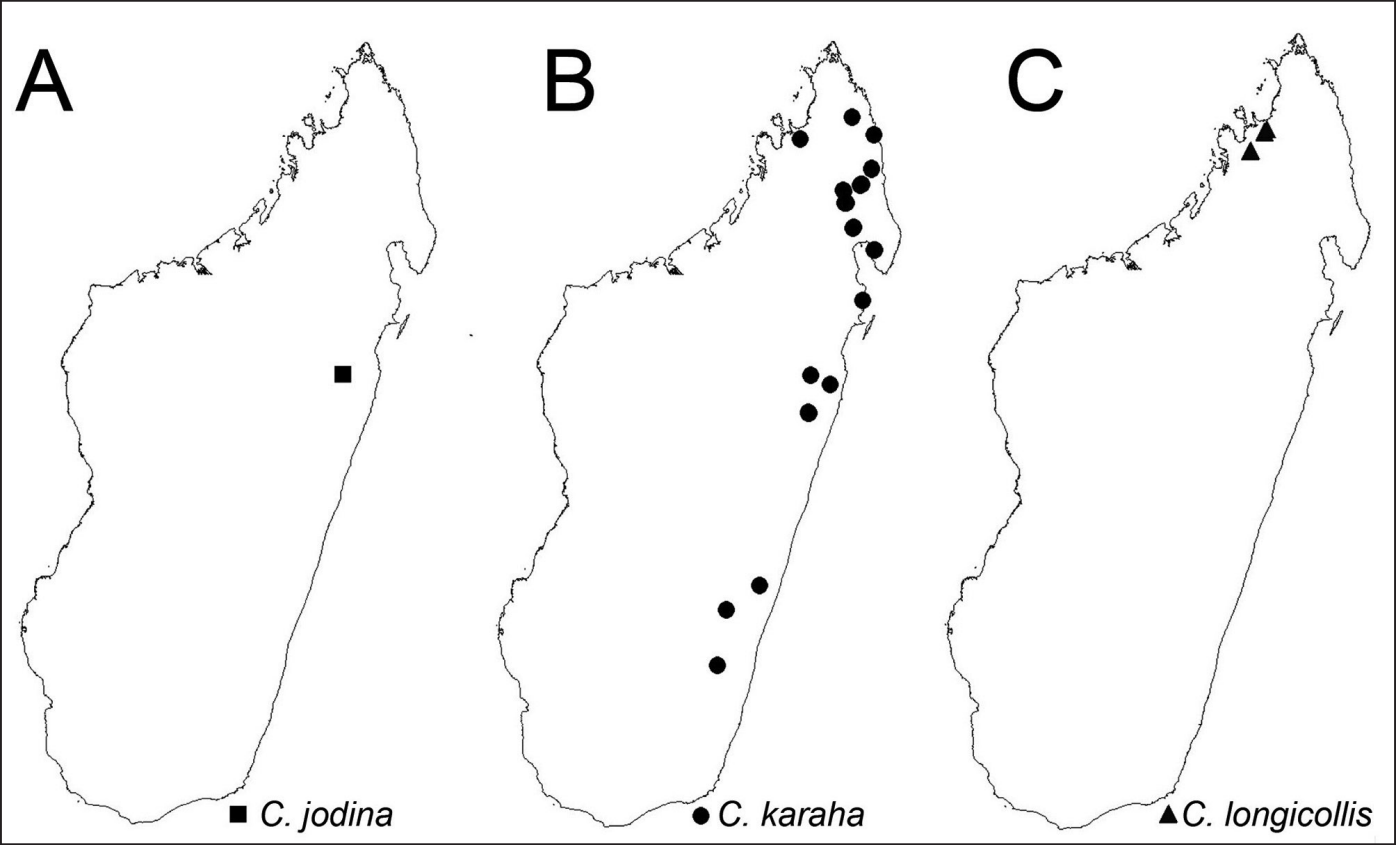
Distribution maps of the three new species. **A** *Camponotus jodina* **B** *Camponotus karaha* **C** *Camponotus longicollis*.

Figures 6A , 7A , 16 , 17 , 18

##### Type material investigated.

Holotype worker: MADAGASCAR, Province Antsiranana, Galoko chain, Mont Kalabenono, -13.63999, 48.67374, 498 m, 15.xi.2013, rainforest, ex rotten log. (B.L. Fisher et al.). Collection code: BLF32079. Unique specimen identifier: CASENT0370614 (CASC).

Paratype workers: Two workers with the same data as holotype but collection code: BLF32133, Unique specimen identifier: CASENT0370620; CASENT0370621 (CASC).

##### Diagnosis.

The following character combination distinguishes *Camponotus longicollis* from *Camponotus karaha* and *Camponotus jodina*: petiole relatively low and nodiform. NOH/CS 0.126 [0.091, 0.181]. Dorsal margin of clypeus weakly convex in lateral view.

##### Description of minor worker.

Head longer than wide, posterior region of head extended into an elongate neck, sides of the head narrowed anteriorly, absolute cephalic size: 2.035 mm [1.74, 2.26] (n = 22). Erect, short, filiform setae present on head. Eyes located posterior to the midlength of head capsule in full-face view, postocular distance vs. cephalic length. PoOC/CL 0.428 [0.364, 0.475]. Frontal carina convex. FR/CS 0.216 [0.191, 0.229]. Scape distinctly surpassing posterior margin by almost half of its length. SL/CS 1.608 [1.434, 1.681]. Prominence on anteromedial clypeal margin projecting as a triangular spur, and dorsal outline of clypeus smoothly convex in profile; masticatory margin with 6 teeth, palps long with respect to head size.

Pronotum weakly undulant. Anterior region of mesonotum (immediately posterior of the promesonotal suture) in profile rising above pronotum. Mesonotum straight and elongate, distinctly compressed laterally anterior to mesothoracic spiracles. MPD/CS 1.473 [1.345, 1.580]. Suberect filiform setae present except on propodeum, which is covered with dense pubescence. Mesothoracic spiracles prominent; propodeal dorsum convex, mesonotum, and dorsum of propodeum meet at a right angle. ML/CS 2.202 [2.106, 2.280].

Petiole low and nodiform. Posterior face of petiole meets its dorsum at a rounded angle. PEW/CS 0.201 [0.170, 0.230].

Entire body dark brown to black; femora and tibiae dark brown, basitarsus of second and third legs light brown, funicular segments light brown becoming dark brown apically. Head and pronotum microreticulate, mesonotum smooth and shiny, propodeum and fore-coxae moderately reticulate-punctate.

##### Description of major worker.

Head suboval, posterolateral sides of head tapering to rear, absolute cephalic size: 2.427 mm [2.418, 2.444] (n=3). Erect, short, filiform setae present on head. Eyes located close to the cephalic midlength. PoOC/CL 0.399 [0.392, 0.409]. Frontal carina convex. FR/CS 0.247 [0.240, 0.256]. Scape surpassing posterior margin by more than one third of its length. SL/CS 1.318 [1.264, 1.345]. Anterior margin of clypeus projecting to an obtuse angle, dorsal outline of clypeus smoothly convex in profile; masticatory margin with 6 teeth, palps long with respect to head size.

Similar appearance as minor worker. Anterior region of mesonotum (immediately posterior to the pro-mesonotal suture) in profile rising above pronotum. Mesonotum straight and elongate, distinctly compressed laterally anterior to mesothoracic spiracles. Suberect filiform setae present, propodeum covered with dense pubescence, pubescence generally sparse elsewhere. Mesothoracic spiracles feebly produced laterally; propodeal dorsum convex, mesonotum and propodeum dorsum meet at a right angle. ML/CS 1.922 [1.853, 1.997].

Petiole low and nodiform. Posterior face of petiole meeting its dorsum at rounded to acute angle. PEW/CS 0.206 [0.190, 0.215].

Overall color black; basal funicular segments yellowish brown and becoming dark apically; basitarsus of second and third legs light brown. Color of filiform setae whitish. Head and pronotum finely reticulate, mesonotum smooth and shiny, clypeus, gena, propodeum and fore-coxae moderately reticulate punctate.

##### Queen.

Unknown.

##### Male.

Unknown.

##### Distribution and biology.

*C. longicollis* has only been collected at two localities in the northwest Sambirano region of Madagascar, Réserve Spéciale de Manongarivo and Mont Kalabenono and Mont Galoko on the Galoko mountain chain (Fig. 18C). It has been collected via litter sifting and hand collecting on a rotten log.

##### Etymology.

This species name is based on the Latin terms for long, “longi”, and collis, “neck”.

##### Additional material examined.

Province **Antsiranana**: Galoko chain, Mont Galoko, -13.58487, 48.71818, 520 m (B.L. Fisher et al.) (CASC); Galoko chain, Mont Kalabenono, -13.63999, 48.67374, 498 m (B.L. Fisher et al.) (CASC); R.S. Manongarivo, 10.8 km 229° SW Antanambao, -13.96167, 48.43333, 400 m (B.L. Fisher et al.) (CASC); R.S. Manongarivo, 12.8 km 228° SW Antanambao, -13.97667, 48.42333, 780 m (B.L. Fisher et al.) (CASC).

## Supplementary Material

XML Treatment for Camponotus imitator

XML Treatment for Camponotus jodina

XML Treatment for Camponotus karaha

XML Treatment for Camponotus longicollis

## Supplementary materials

Supplementary material 1 Ratios of morphometric data for all specimens Data type: species morphometric data This dataset is made available under the Open Database License (http://opendatacommons.org/licenses/odbl/1.0/). The Open Database License (ODbL) is a license agreement intended to allow users to freely share, modify, and use this Dataset while maintaining this same freedom for others, provided that the original source and author(s) are credited.Nicole Rasoamanana, Sándor Csősz, Brian L. Fisher
